# Polyurethane-Based Nanocomposites for Regenerative Therapies of Cancer Skin Surgery with Low Inflammatory Potential to Healthy Fibroblasts and Keratinocytes In Vitro

**DOI:** 10.1021/acsomega.3c01663

**Published:** 2023-10-02

**Authors:** Maciej Mrówka, Joanna Lenża-Czempik, Anahit Dawicka, Magdalena Skonieczna

**Affiliations:** † Department of Material Technologies, Faculty of Material Engineering, Silesian University of Technology, Krasińskiego 8, 40-019 Katowice, Poland; ‡ Material Innovations Laboratory, Silesian University of Technology, Krasińskiego 8, 40-019 Katowice, Poland; § Krahn Chemie Polska Sp. z o.o., Marcelińska 90, 60-324 Poznań, Poland; ∥ Biotechnology Center, Silesian University of Technology, Krzywoustego 8, 44-100 Gliwice, Poland; ⊥ Department of Systems Biology and Engineering, Silesian University of Technology, Akademicka 16, 44-100 Gliwice, Poland

## Abstract

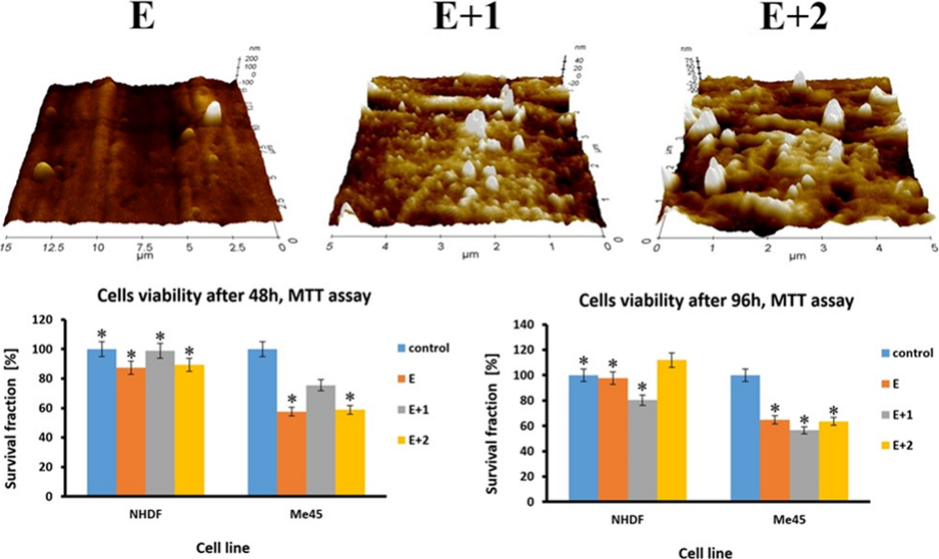

Nanocomposites based on thermoplastic polyurethanes (TPUs) filled with halloysite nanotubes (HNTs) were studied for their physicochemical and biological properties. Nanocomposites containing halloysite nanotube filler contents of 1 and 2% (E+1 and E+2), respectively, were obtained by extrusion. The newly formed E+1 and E+2 nanomaterials exhibited better flexibility and similar thermal properties compared to neat polyurethane. The use of atomic force microscopy (AFM) and differential scanning calorimetry (DSC) thermogram analysis showed that the distribution of halloysite nanotubes in the polymer matrix is more evenly dispersed in the E+1 nanomaterial, where the grains in the E+2 nanomaterial have a greater tendency to form agglomerates. Mechanical tests have shown that nanocomposites with the addition of HNT are characterized by a higher stress at break and elongation at break compared to neat TPU. The results of cytotoxicity tests suggest that the nanocomposite materials express lower toxicity to normal HaCaT and NHDF than to cancer Me45 cells. Further studies showed that the tested materials induced the expression of proinflammatory interleukins IL6 and IL8 in normal cells, but their overexpression in the cancer cell line resulted in cytostatic effects and proliferation reduction. Such a conclusion suggests the possible application of tested materials for regenerative therapies in cancer surgeries.

## 1 Introduction

Thermoplastic polyurethanes (TPUs) have numerous applications in medicine due to their physicochemical properties, such as elasticity, thermal resistance, low abrasion, and resistance to aging under physiological conditions, with a simultaneous lack of toxicity to the human body.^1−5^ In cardiac surgery, TPU primarily produces heart valve leaflets and artificial heart components.^6,7^ TPU is also used to make blood bags, apparatuses for extracorporeal blood oxygenation, breast implants, catheters, dental floss, elements of oxygen masks, bone adhesives, artificial blood vessels, stomach balloons, components of esophageal prostheses, and tendon prostheses.^8−15^

Various fillers, particularly those of natural origin, have been introduced to improve the mechanical, thermal, and biological properties of TPUs. Nanocomposites with a polymer matrix, such as halloysite nanotubes (HNTs), have many applications in manufacturing. Halloysite, a natural, inorganic material, is physically and chemically analogous to kaolinite (Al_2_Si_2_O_5_(OH)_4_·*n*H_2_O). Structurally, HNTs range from 200 to 1000 nm in length and 20 to 200 nm in inner diameter.^16−19^

HNTs have found various applications in medicine. For example, HNTs comprise a promising drug delivery carrier due to their biocompatibility, low toxicity, and ability to carry active agents.^20−26^ HNTs are already in use as a drug delivery vector for a variety of pharmaceutical products, including anticancer drugs (e.g., camptothecin,^27^ paclitaxel,^28^ doxorubicin,^29,30^ curcumin,^31^ quercetin,^32^ and 5-fluorouracil^33^), antibiotics (i.e., tetracycline^34^ and ciprofloxacin^35^), analgesics (i.e., diclofenac sodium^36^), antihypertension (i.e., polydopamine^37^), anti-inflammatory drugs (i.e., ibuprofen^38,39^ and aspirin^40^), and therapeutic nucleic acids.^41^

HNTs have recently gained considerable attention as a new type of nanoadditive for enhancing the mechanical, thermal, crystallization, and fire performance of thermoplastic polymers, such as polycatides,^42^ poly(butylene succinate),^43^ polypropylene,^44,45^ polyamide-6,^46^ and thermosets, such as epoxy.^47^

HNTs have allowed TPUs to gain new desirable properties among the various types of fillers available. TPU-HNT nanocomposites were effectively prepared and used in a variety of applications in both engineering and biomedical fields.^48^ HNTs have enhanced the mechanical, thermal, crystallization, and fire performances of thermoplastic polymers. A few reports on the mechanical properties of HNT-reinforced TPU mainly focus on the mechanical and thermal properties of the tested nanocomposites. TPU-HNT nanocomposites showed an increased tensile modulus, stress at break, and elongation at break in samples containing only small percentages of HNTs.^49−53^

So far, little research has been conducted on the properties of TPU-HNTs for the antibacterial effects of TPU–HNT nanocomposites,^54^ and the cytotoxicity of TPU–HNT has not yet been investigated. The objective of this study is to investigate the biological and physicochemical impacts of TPU–HNT in both standard and cancerous skin cells. It is hoped that these findings may open promising new applications for these materials, specifically in regenerative therapies of skin cancer surgery, where the nontoxic abilities of healthy fibroblasts and keratinocytes are needed.

## 2 Materials and Methods

### 2.1 Materials

The research was conducted on nanocomposites based on thermoplastic linear TPUs filled with HNTs. Halloysite nanoclay has a tube morphology of 30–70 nm in diameter and 1–3 μm in length. Elastollan 1185A is a linear TPU purchased from the BASF company that was used to create samples for testing.

### 2.2 Nanocomposite Preparation

Before extrusion, Elastollan 1185A was dried in a convection oven for 3 h at 110 °C. Two batches of test nanocomposites were compounded, composed of thermoplastic polyurethane filled with HNT at 1 and 2% weight fractions and labeled [E+1] and [E+2], respectively. A third batch of neat TPU, designated as [E], was also compounded. The nanocomposites were extruded using a Leistritz ZSE 27 HP corotating twin-screw extruder with zone temperatures ranging from 100 to 175 °C, a mass temperature of 180 °C, a pressure at the head of 4.5 MPa, and 270 rpm screw rotation with 12 kg/h efficiency.

### 2.3 Sample Preparation

**Figure 1:**
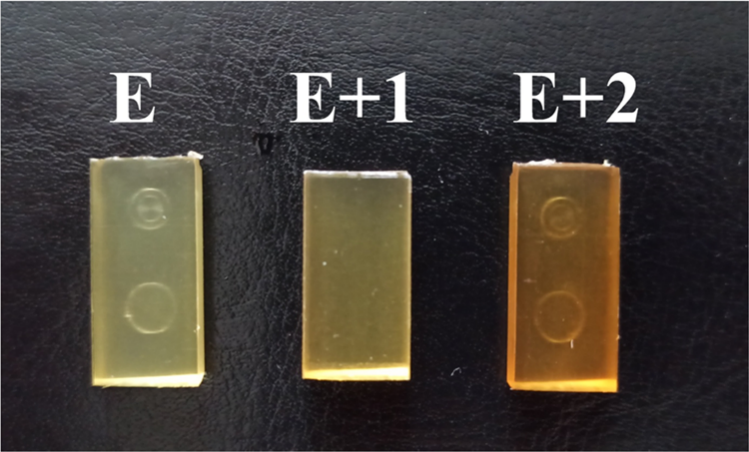
Samples of tested materials: E (neat TPU), E+1, and E+2 nanocomposites.

Materials were dried in a convection oven for 3 h at 110 °C before injection molding. Samples for all tests were prepared by injection molding on an Arburg Allrounder 270-210-500 machine at 180–195 °C and 90 MPa. The injection molding process yielded cuboidal samples with dimensions of 10 × 4 × 80 mm^3^. The cuboids were then cut on a microtome to obtain 500 μm thick samples for subsequent tests. The injected models are shown in Figure 1.

### 2.4 Scanning Transmission Electron Microscopy (STEM)

The structure and morphology of the nanocomposites were analyzed by scanning transmission electron microscopy (STEM). For STEM observations, specimens were prepared by the focused ion beam (FIB) technique using a SEM/Ga-FIB Helios Nano-Lab 600i microscope (FEI, Hillsboro, OR). The measurements were performed with an S/TEM TITAN 80-300 microscope with an energy-dispersive X-ray spectrometer (EDS). High-angle angular dark field (HAADF) images were collected with a 24.5 mrad probe semiangle, with a HAADF detector range of 47–200 mrad.

### 2.5 Atomic Force Microscopy

Contact-mode atomic force microscopy (AFM) was used to characterize the surface topography and confirm the particle size of HNTs in the E+1 and E+2 nanocomposite samples.^55^ The measurements were obtained using an XE-100 microscope XE-100 (ParkSystems). Data were collected in the air at room temperature.

### 2.6 Differential Scanning Calorimetry (DSC)

The thermal stability of the obtained materials was evaluated by differential scanning calorimetry (DSC) using a TG/DSC device (Mettler Toledo, Columbus, OH). Samples (∼5 mg) were heated under a nitrogen atmosphere from −70 to 500 °C at 10 °C/min.^55,56^ The glass-transition temperature (*T*_g_) parameter was evaluated based on the derived DSC curves.

### 2.7 Contact Angle Measurement

Hydrophilic surfaces are desirable for biomedical applications. Because HNT incorporation impacts the hydrophilicity of the TPU, surface wetting of the samples was measured by contact angle measurements using a Surftens Universal Measuring Instrument (OEG GmbH) equipped with a thermal chamber. Static contact angles of water were calculated using Surftens 4.3, Windows image processing software for digital images. For each sample, three independent 1.5 μL water droplets were applied. The mean value was averaged over 10 measurements.

### 2.8 Mechanical Tests

The tensile test was measured by EN ISO 527-1 on a tensile machine, Instron 4465 (Instron, Norwood, MA) equipped with a mechanical contact extensometer. The test speed was 50 mm/min. A sample population of 5 was used for all experiments. Using the tensile test results for each of the tested sample populations, the fracture stress and elongation were determined along with the tensile modulus. Hardness measurements of the tested materials were carried out using a hardness durometer Shore A type Zorn (Zorn Instruments GmbH & Co., Hansestadt, Germany). The Shore A hardness test was completed by ISO 686. Five measurements were taken for each composite while maintaining a distance of at least 10 mm from the sample edge and between individual measurements.

### 2.9 Biological Evaluation (Cytotoxicity, Microscopic Viability, and Proinflammatory Assays)

#### 2.9.1 Cell Culture

Cytotoxicity tests of the nanomaterials were carried out on normal human dermal fibroblasts (NHDF-Neo, Lonza, Poland), keratinocytes (HaCaT; from CLS collection, Germany), and human malignant melanoma (Me45) cell lines. Human malignant melanoma cells (Me45) established from a lymph node metastasis of primary skin melanoma were obtained from the collection of the Maria Skłodowska–Curie National Institute of Oncology, Gliwice Branch (Poland), and cultured as previously reported.^57,58^ The cell lines were cultured in a monolayer in DMEM-F12 (PAA, U.K.) supplemented with 10% fetal bovine serum (Eurx, Poland) and 1% antibiotic antimycotic solution (Sigma) under standard conditions.

#### 2.9.2 MTT Cytotoxicity Assay

Cell viability was assessed using the MTT test (3-[4,5-dimethylthiazol-2-yl]-2,5-diphenyltetrazolium bromide). Cells were inoculated in Petri dishes at the rate of 10^5^ cells per well. Cells were seeded on tested materials (diameter of 1 cm^2^) and incubated for 24 and 72 h at 37 °C in a humidified atmosphere saturated with 5% CO_2_. After the incubation, the culture medium was removed and replaced with trypsin for cell collection (Sigma). After trypsin neutralization, the cell suspension was collected and centrifuged (2000 rpm, 3 min, room temperature), and the cell pellet was suspended in the MTT solution (50 μL, 0.5 mg/mL in RPMI 1640 without phenol red, Sigma). After 3 h of incubation, the MTT solution was removed, and the resulting formazan was dissolved in isopropanol: HCl. Absorbance at 570 nm was measured spectrophotometrically using a microplate spectrophotometer (Epoch; BioTek). The experiment was carried out in three independent replicates.

#### 2.9.3 Live Cell Imaging

Live observations were performed on healthy keratinocytes (HaCaT), fibroblasts (NHDF), and melanoma Me45 cell lines using a JuLI_FL apparatus (NanoEntek). Cells were cultured on 3 cm diameter Petri dishes (Beckton Dickinson), with an initial seeding density of 5 × 10^3^ cells in 5 mL of fresh DMEM-F12 medium. To monitor cell proliferation, the control and untreated cells were directly observed in the transit channel after 24 h. Finally, the images were recorded after 96 h by the automated analyzer in the transit channel for cell detection and confluence counting. Image preprocessing, cell counting, and viability analysis were conducted according to the procedure for an automatic live confluence assay, a JuliTmSTAT Cell analysis software version 2.0.1.0 by NanoEnTek Inc. Sets of images taken from control and treated cells were analyzed, and the confluence from discriminated areas was expressed as % value in comparison to the control (where 0% means no cells in the observed area). The procedure and scheme of image processing are presented in Table 1.

**Table 1:**
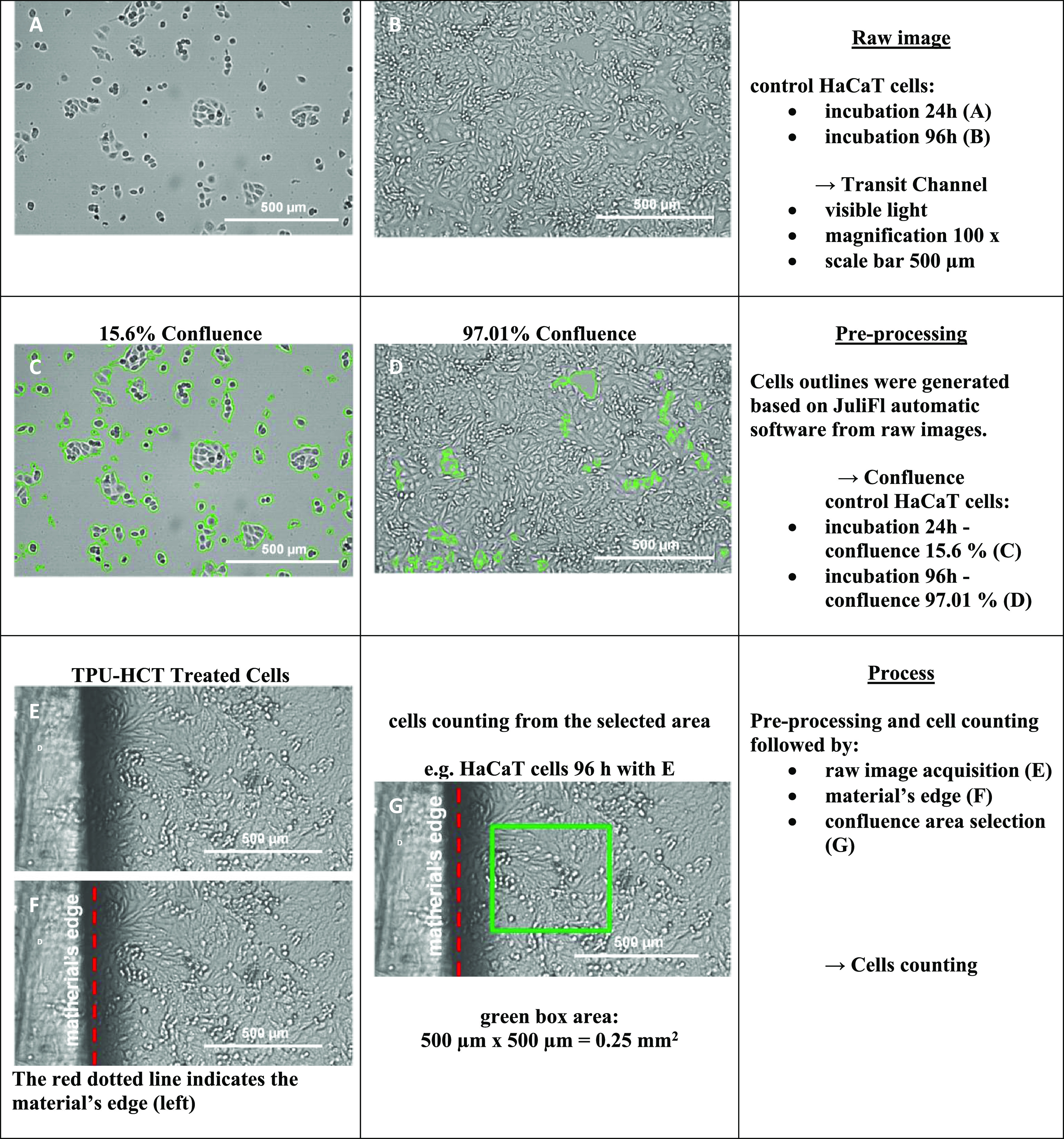
Scheme of an Automated Procedure of the Image Analysis for Cell Confluency and Cell Number Counting in the Control and TPU-HNT-Treated Cells after 24 h (A) and 96 h (B) of Incubation with Keratinocytes HaCaT (Raw Image); Samples of Cell Counting and Confluence Analysis after 24 h (C) and 96 h (D)—Image Preprocessing; Sampling of Defined Areas in Control and Treated Cells (E–G)—Processing

#### 2.9.4 Confocal Microscopy Imaging

To perform confocal microscopy observations, the NHDF cells were seeded on the surfaces of tested materials, previously sterilized with UV radiation or a control polystyrene plate (Sarstedt, Numbrecht, Germany). After incubating for 96 h, the cells were fixed using 70% methanol solution in deionized water for 10 min at room temperature, washed with water, and stained with DAPI dye (Invitrogen, Waltham, MA) for nucleus visualization. Cell signals grown directly on tested materials were captured using confocal microscopy and an Olympus FluoView FV1000 apparatus (Olympus LS, Tokyo, Japan).

#### 2.9.5 Real-Time PCR Proinflammatory Gene Expression Assay

Gene expression assessments of proinflammatory interleukins IL6 and IL8 (RT-qPCR) were performed for control cells and after 48 h of incubation with tested materials for normal NHDF and HaCaT cell lines. After cells were trypsinized and collected, the total RNA was isolated using the phenol-chloroform extraction method, by the total RNA isolation kit (A@A Biotechnology). The efficiency of RNA isolation was assessed spectrophotometrically, and amplification of IL6 and IL8 genes (proinflammatory genes) was performed using commercially available kits (Real-Time 2xPCR Master Mix SYBR A; A@A Biotechnology) and pairs of primers (Genomed): (i) IL6 reverse: AGATCACCTAGTCCACCCCC; IL6 forward: GTTCTGCCAAACCAGCCTTG; (ii) IL8 reverse: ACCAAGGCACAGTGGAACAA; IL8 forward: GGTGCAGTTTTGCCAAGGAG; (iii) reference RPL41 reverse: ACGGTGCAACAAGCTAGCGG; reference RPL41 forward: TCCTGCGTTGGGATTCCGTG. The quantitative PCR reaction, preceded by reverse transcription (NG dART RT kit, EURx), was performed by using a CFX96 Touch Real-Time PCR Detection System thermocycler (Bio-Rad). The thermal profile of the reaction was as follows: (1) 50 °C, 2 min, (2) 95 °C, 4 min, (3) 54 cycles of 95 °C, 45 s; 52.3 °C, 30 s.; fluorescence reading; (4) 72 °C, 5 min; (5) melting curve from 52 to 92 °C (every 0.5 °C at 5 s); and (6) incubation for every sample at 4 °C. The calculation of the standardized value of the relative gene expression level in an unknown sample was performed concerning controls, following Livak’s formula^59^
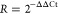


where *R* is the ratio of the relative gene expression between target and reference genes, Ct is the quantification cycle (Ct), and ^–ΔΔCt^ is the difference between the quantification cycle (Ct) of target and reference genes.^59^

#### 2.9.6 Statistical Analysis

Three separate experiments express the results as means ± SD. Statistical significance was calculated with a *t* test, and in comparison to the control, the important changes, when the *P*-value <0.05, were indicated on charts with a star (*). Data were analyzed using MS Office ver. 2.5.0 and MS Excel 2010.

## 3 Results and Discussion

### 3.1 Scanning Transmission Electron Microscopy (STEM)

**Figure 2:**
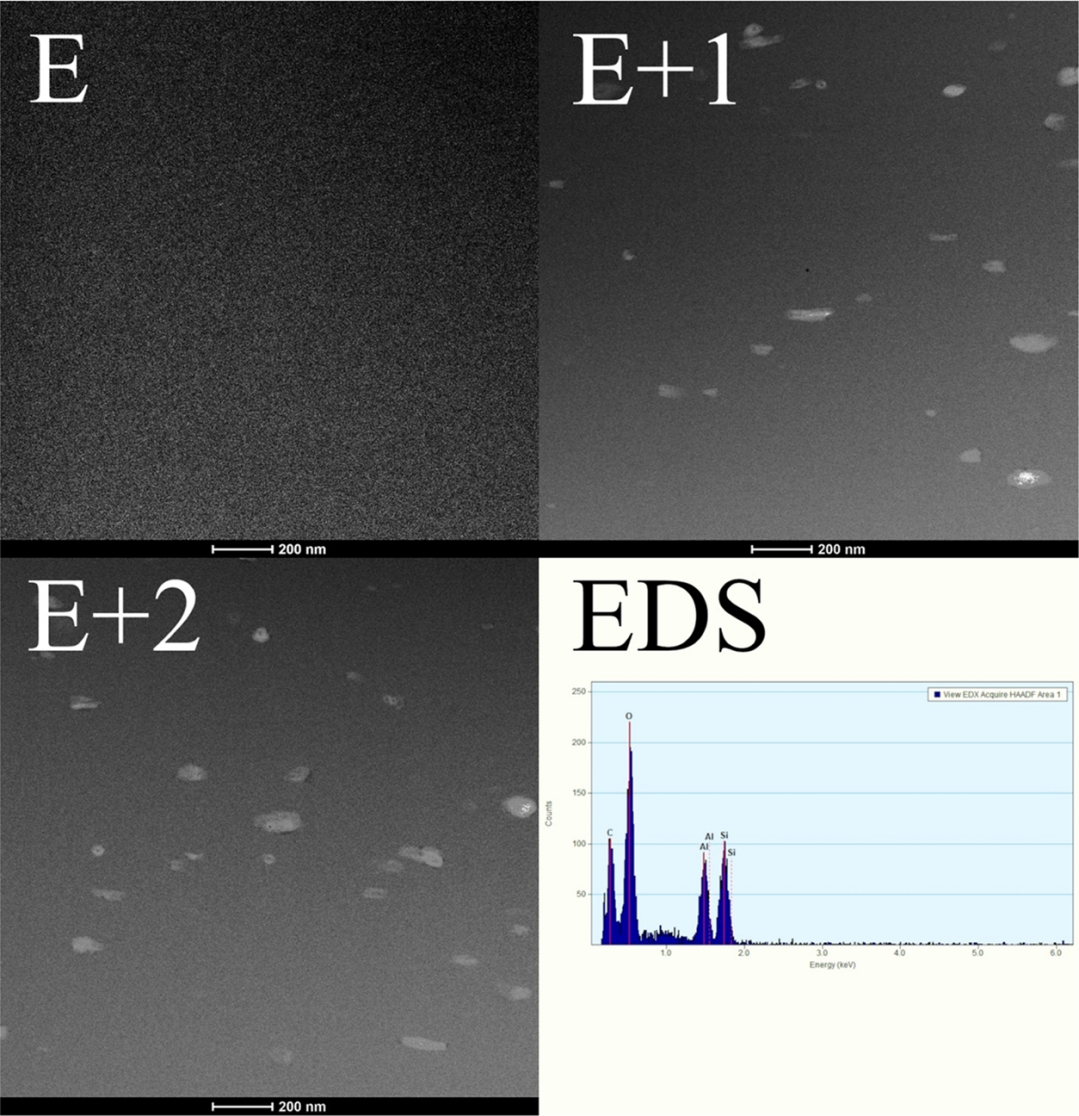
STEM-HAADF images of the tested materials: E, E+1, E+2. Energy-dispersive (EDS) spectra were obtained for a single halloysite tube in a polyurethane matrix.

The HNTs (bright tubular region) are evenly embedded in the polymer matrix (dark area). The composite components are easily distinguishable due to the high-angle annular dark field (HAADF) detector, which registers the electrons passing through the sample and scattering at a high angle (Rutherford scattering). As a result, the recorded signal intensity is proportional to the atomic number of Z of the dominant element in each specimen area (Z-contrast). As halloysite nanotubes contain additional Al and Si (EDS), with a higher *Z* value than the elements in the matrix material (mainly C and O), they are seen as brighter than the polymer matrix. Most of the HNTs are homogeneously dispersed. The STEM image is only a two-dimensional projection of the three-dimensional structure of the composite, which was additionally prepared in the form of a thin foil (approximately 100 nm thick). However, the privileged orientation of the nanotubes is noticeable for all investigated materials (in the presented images, they have a vertical direction). The HNT length does not exceed 500 nm, and its diameter does not exceed 100 nm (Figure 2).

### 3.2 Atomic Force Microscopy

**Figure 3:**
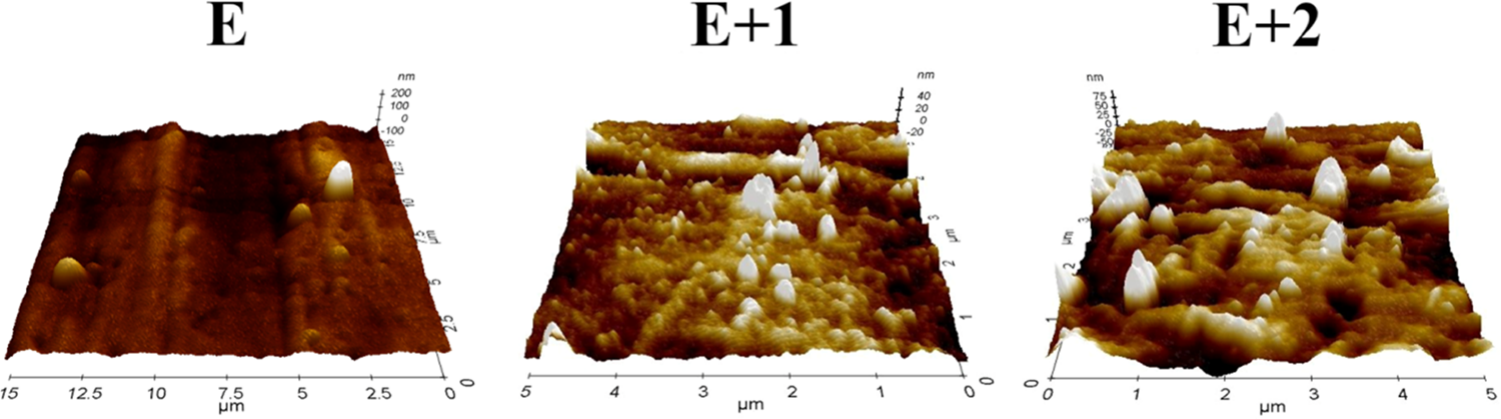
AFM surface topography of E, E+1, and E+2 materials.

Figure 3 presents the AFM 3D images of the material surface. The surface topography of the produced composites was examined using the Park Systems AFM XE-100 AFM, operating in a noncontact mode with integrated XEI software. Additionally, as part of the quantitative presentation of the surface topography, the roughness coefficients of the sample surfaces were estimated: the mean arithmetic deviation from the mean line (*R*_a_) and the surface roughness coefficient, rough mean square (RMS).

The topographic image of the surface of a pure TPU sample marked as E shows surface irregularities in the form of undulations with few granular structures ∼1 μm in size (which may be polymer aggregates). However, the sample surface roughness coefficients indicate that the degree of surface heterogeneity is insignificant as this type of sample surface condition is typical of the manufacturing method used.

Topographic images of composite sample E+1 and E+2 surfaces show numerous granular structures of various shapes and sizes below 1 μm. This suggests the presence of a nanotube filler material and its agglomerates on the sample surface. The occurrence of nanostructure agglomerates on the surface of polymer matrix composites is a consequence of the natural tendency of nanostructures to agglomerate and the production method used. In the case of the E+2 sample, a higher proportion of larger granular structures was observed compared to that of the E+1 sample. This is probably a consequence of the more significant proportion of filler in the polymer matrix. The RMS and Ra ratios confirm the observed relationship.

### 3.3 Differential Scanning Calorimetry (DSC)

**Figure 4:**
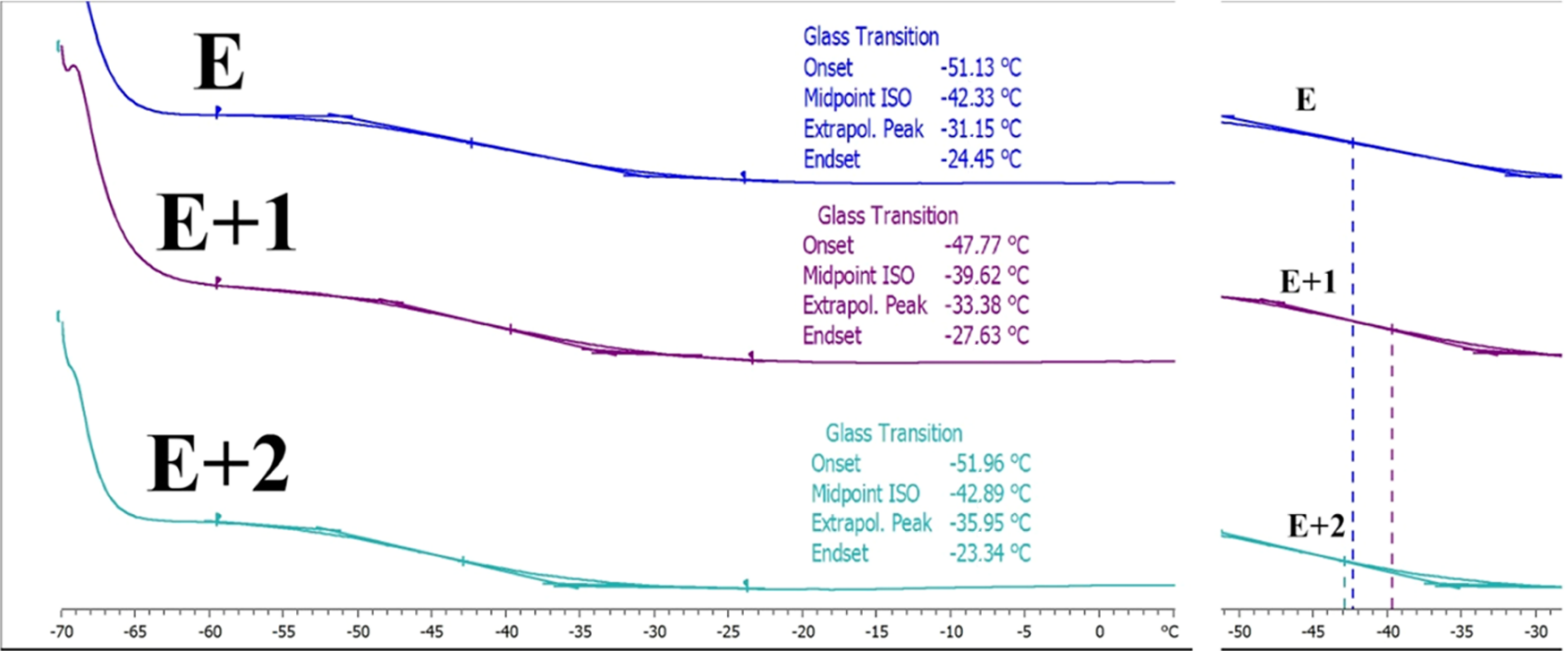
DSC curves for the materials tested.

Knowledge of the thermal behavior of neat TPU, E+1, and E+2 was required to understand the molecular structure of new nanocomposites. DSC was used to measure the glass-transition temperature (*T*_g_) of pure TPU (E) and TPU with HNT (E+1, E+2) (Figure 4). The *T*_g_ peak for pure TPU appears at −42.33 °C. The presence of nanoparticles in pure TPU caused an interaction between the TPU chains and the HNT nanoparticles. These interactions caused changes in the movement of polymer chains and influenced the *T*_g_. The chemical interactions between TPU chains with HNTs led to a slight increase in the *T*_g_ of soft RPU segments to −39.62 °C with 1% filler content (E+1). However, the opposite trend is observed when the HNT filler content increased to 2% (E+2). The *T*_g_ of the E+2 sample was −42.89 °C. The magnitude of the changes in *T*_g_ compared to pure TPU was greater for E+1 samples. This suggests that TPU and HNT interaction is lower in E+2 samples, which could be explained by the poor HNT distribution throughout the TPU. Another reason for this decreased *T*_g_ can be attributed to the accumulation and aggregation of HNTs, which is more significant in E+2 samples compared to E+1 samples according to AFM surface topography results.

### 3.4 Contact Angle Measurement

**Figure 5:**
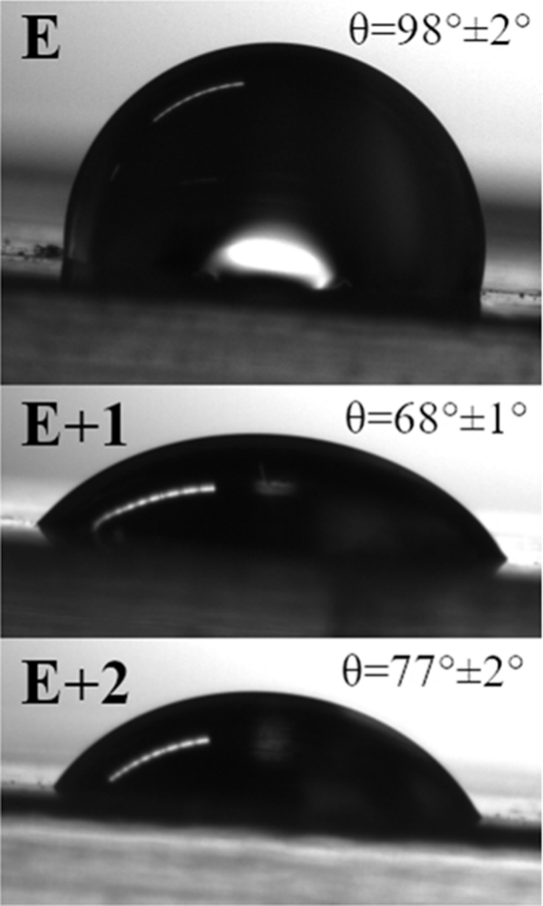
Summary of contact angle measurements with H_2_O.

Biological interactions, such as cell adhesion, are strongly correlated with the surface energy of the implant material, which can be quantitatively characterized through the contact angle method. The introduction of the HNT described above significantly influences the hydrophilic properties of TPUs. This modification can be quickly followed by contact angle measurements using H_2_O droplets deposited on unmodified and modified (E+1, E+2) TPU (Figure 5). The entire TPU sample proved relatively hydrophobic, with a water contact angle measurement of θ = 98 ± 2°, in agreement with previously reported data.^60^ HNTs can affect the hydrophobic properties of the TPU structure, however, and the water contact angles for the modified samples E+1 and E+2 were 68 ± 1° and 77 ± 2°, respectively. Therefore, the contact angle of TPU decreases when adjusted with HNT. These results are significant because of their impact on cell attachment. As reported elsewhere, fibroblasts adhere and differentiate more on surfaces with greater hydrophilicity.^60^ This can be explained by the interaction of the polar group’s OH in the halloysite molecular structure with fibroblast cells. Specifically, the hydroxyl groups enhance fibronectin absorption and exposure of cell adhesive domains related to focal adhesion and cell growth and increase the differentiation of fibroblasts.^61^

### 3.5 Mechanical Tests

**Figure 6:**
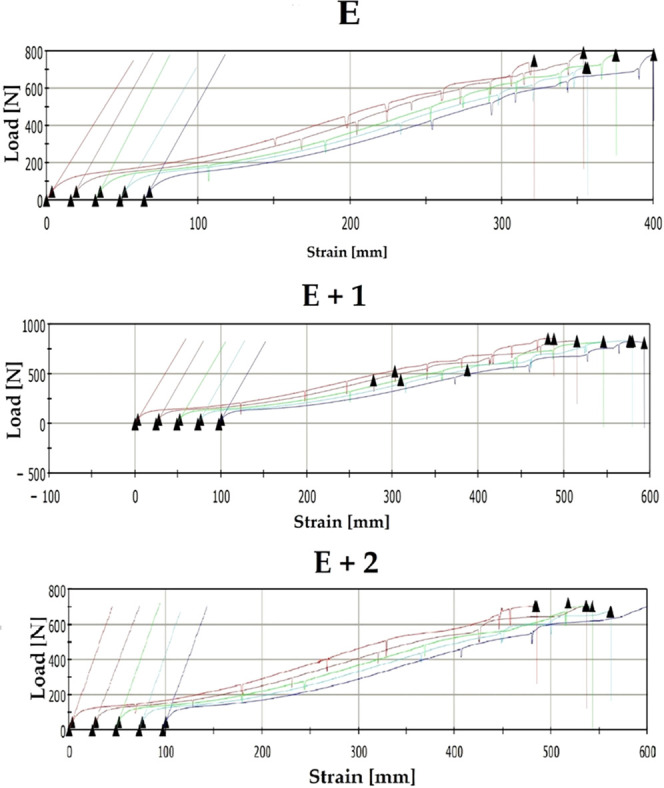
Load–strain curves of the materials.

Static tensile tests were performed on samples composed of unmodified TPU [E] and the nanocomposites [E+1] and [E+2]. The load–strain curves of the materials are shown in Figure 6.

The materials were characterized by measuring the Young modulus, stress at break, elongation at break, and hardness. The results are presented in Table 2.

**Table 2:** Results of Mechanical Tests of the Obtained Materials

	E	E+1	E+2
Young’s modulus [MPa]	26.35 ± 2.36	26.04 ± 0.49	27.28 ± 0.79
stress at break [MPa]	28.31 ± 3.89	34.87 ± 1.77	33.69 ± 0.93
elongation at break [%]	723.6 ± 32.79	979.4 ± 59.76	1085.2 ± 18.96
hardness [ShA]	86.8 ± 0.017	88.2 ± 0.003	88 ± 0.006

For unmodified TPU [E], the measured Young modulus was 26.35 MPa, while for [E+1], a decrease of 1% (26.04 MPa) was measured. For the [E+2] nanocomposite, the Young modulus increase compared to [E] is 3.5% (27.28 MPa). The mechanical testing results for these materials indicate that the Young modulus does not change with an increasing HNT concentration. The obtained results differ significantly from those presented in the literature. The publication^49^ reported a 32% increase in the Young modulus for nanocomposites containing 1% HNT mass content. Similarly, publication^50^ reported a 40% increase in the Young modulus for nanocomposites containing 3.7% HNT.

For the nanocomposite [E+1], a 12% increase in stress at break compared with that of [E] was measured (i.e., 28.31 MPa compared with 34.87 MPa). For [E+2], an 11% increase compared with [E] (i.e., 28.31 MPa vs 33.69 MPa) was also read. Therefore, the stress-at-break values for both nanocomposites should be treated as equal. The literature reports different relationships for nanocomposites filled with some form of HNT. For instance, publication^51^ showed an increase in stress at break by 37% for a nanocomposite containing 1% HNT. Publication^49^ describes a 44% increase in pressure at break for a nanocomposite with 1% HNT content. Similarly, the authors^48^ presented studies in which a nanocomposite containing 1% HNT showed a 43% increase in stress at break compared with the TPU matrix. Additionally, 2% additions of HNT to the TPU matrix have been reported to increase the pressure at break by 26%.^53^

[E+1] showed a 35% increase in elongation at break compared with [E] (i.e., 723.6–979.4%), while [E+2] showed a 50% increase in elongation at break compared with the matrix (i.e., 723.6–1085.2%). The elongation at break values in the literature are significantly higher than those described in this paper. In publication,^48^ a 1% HNT-TPU nanocomposite resulted in a 144% increase in elongation at break compared with native TPU. In publications studying similar nanocomposites with 2% HNT content, 67%^33^ and 100%^53^ increases in elongation at break were measured compared to TPU. The differences in these results can be attributed to the adopted method of producing nanocomposites or to the materials used in the study. The methods described in the publications are laboratory methods in which processes for obtaining nanocomposites are carried out on a small scale, while this article relies on industrial production methods (i.e., twin-screw, 10-zone extrusion). The source materials also play a significant role. The literature describes nanocomposite production using commercially available TPU, while the source materials for this study use chemically modified TPU to reduce hydrophobicity and improve HNT adhesion.^48–51,53^

The hardness of unmodified TPU was measured at 86.8 ShA, and the hardness values of the nanocomposites were as follows: [E+1] 88.2 ShA and [E+2] 88 ShA. A slight ∼2% increase in hardness was observed for the nanocomposites compared with the native TPU. However, considering the accuracy of the Shore A hardness test, it can be concluded that HNT additions to the TPU matrix do not significantly impact hardness.

### 3.6 MTT Cytotoxicity Assay

**Figure 7:**
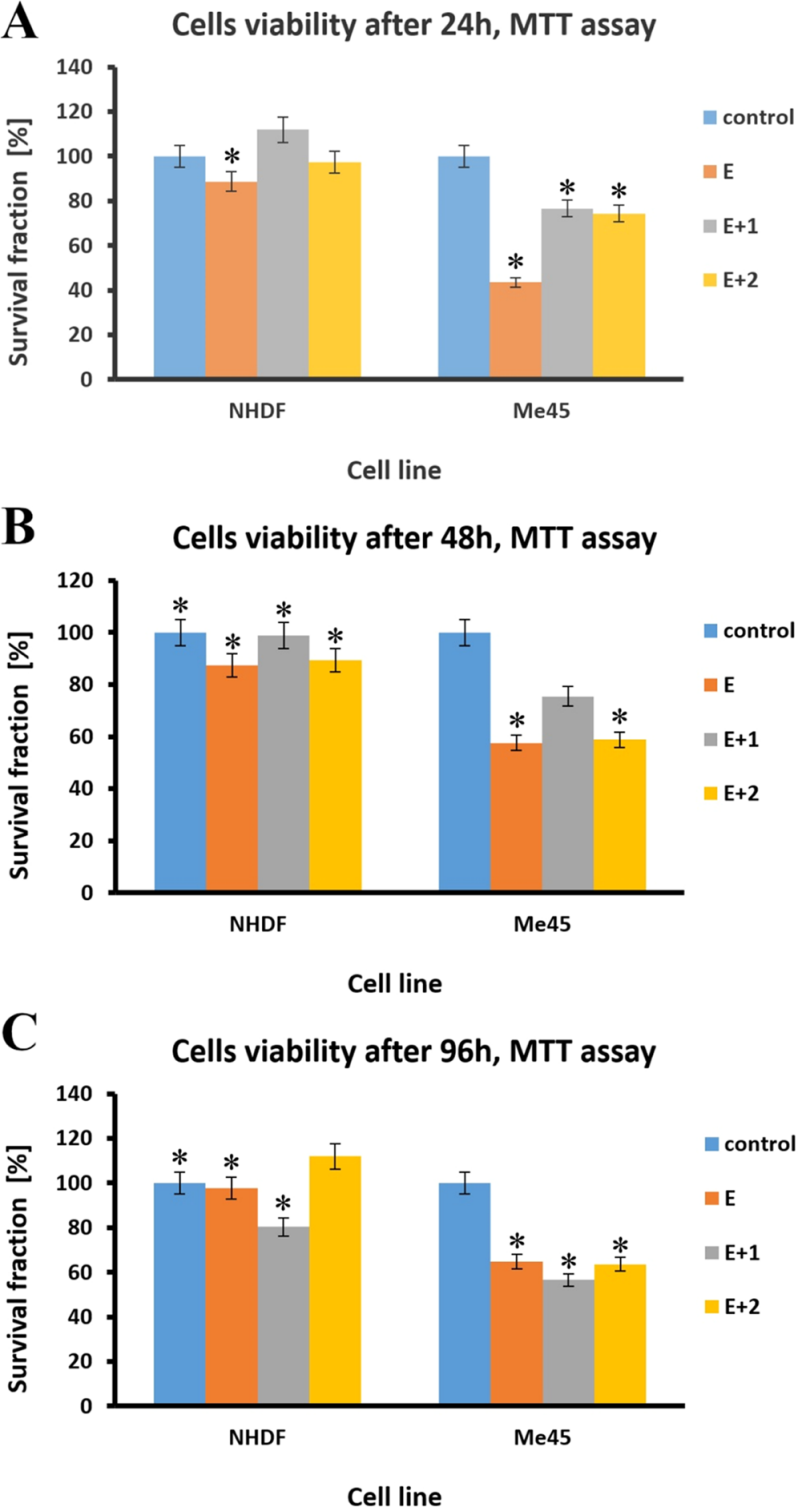
Cytotoxicity of the tested materials relative to the NHDF and Me45 cell lines after 24 h (A), 48 h (B), and 96 h (C) measured in terms of survival fraction [%].

The cytotoxicity results of the tested materials against the NHDF and Me45 cell lines after 24, 48, and 96 h of TPU–HNT incubation are presented in Figure 7A–C. This test aimed to assess the tested materials’ toxicity for their potential use as biomaterials for regenerative medicine. Toxicity is defined as a material feature contributing to disorder, inflammatory state, or death of human cells. These changes are primarily seen in abnormal cell metabolism, and the MTT test can assess the degree to which this phenomenon is present. The results are presented as survival fraction (%) graphs based on the incubation time of cells with the tested material.

No toxic effects were seen on fibroblasts after NHDF cells were incubated with the test materials for 24 h. The average cell viabilities for E, E+1, and E+2 were 89, 112, and 97%, respectively. At the same time, the viability of Me45 cells decreased relative to that of the control. The survival fraction of Me45 cells decreased by 57% compared to the control; untreated cells after 24 h of incubation with E. E+1 and E+2 samples exhibited 23 and 26% drops in survival fraction, respectively, compared to the control samples.

After 48 h, the cell viabilities of the NHDF line against E, E+1, and E+2 were 87, 99, and 89%, respectively. At the same incubation time, a further decrease in the cell viability of the Me45 line was observed. After 48 h, a 42% decrease in cell fraction was observed for cells incubated with material E, a 24% decrease for cells incubated with material E+1, and a 41% decrease for cells incubated with E+2 concerning control cells.

After 96 h of incubation of materials with NHDF cells, cell viabilities were 98, 81, and 112% for cells incubated with E, E+1, and E+2, respectively, concerning control cells. After 96 h, a consistent decrease in the viability of the Me45 tumor cells was observed. The viability of tumor cells decreased by 35, 43, and 36% compared to fibroblasts after 96 h of incubation with E, E+1, and E+2, respectively.

Similar trends were observed for all incubation periods (24, 48, and 96 h). For fibroblasts, the viability of the tested materials was close to the control, with untreated cell survival fraction values. Slight decreases in survival fraction can be explained by cellular stress caused by the appearance of a foreign element in the medium, which was the sample being tested (i.e., E, E+1, or E+2), and the partial, mechanical damage to some cells during sample introduction in culture. The viability of Me45 tumor cell lines was decreased at all test times. The most significant decrease in cell viability in Me45 cells was 43% after 48 h of incubation with E+1. HNT incorporation into the TPU matrix did not affect the antitumor properties of the materials because, after 48 and 96 h of incubation, cell mortality of E was comparable to TPU with the highest concentration of HNTs.

These results indicate the lack of cytotoxicity in normal human fibroblasts to the tested materials after incubation times up to 96 were measured by the MTT test. It should be emphasized that E, E+1, and E+2 samples exhibited antitumor activity with a simultaneous lack of cytotoxicity to noncancerous cells.

### 3.7 Microscopic Viability Observations

Live long-term microscopy enabled the observation of proliferation potential and cell viability assessment during 42 h of incubation with the tested materials. Compared with the untreated control cells (100%), single-cell counting from a defined surface was possible. Normal skin HaCaT and NHDF cells and Me45 cells responded to the presence of the tested materials. Typically, cells responded with proliferation inhibition and decreased cell viability (Table 3). For E and modified materials E+1 and E+2, cell proliferation was inhibited. The most antiproliferative action was reported in the Me45 cancer cell line, where the calculated confluence was nearly the lowest reported (Table 3C). MTT results for NHDF fibroblasts (Figure 7) did not correlate with microscopic observations for the E+2 incubation. The mitochondrial activity evaluated by the MTT assay was still comparable to the untreated controls. However, the proliferation and cell number could be lower due to the cytostatic effect of tested materials. The observation of long-term and live cells indicates healthy cells’ fitness, condition, and proper morphology and inhibition of proliferation for Me45 cells. Cytotoxic and cytostatic effects against the cancer Me45 cell line were confirmed for the tested materials, and selectivity against cancer cells was visible (Figure 7 and Table 3).

**Table 3:**
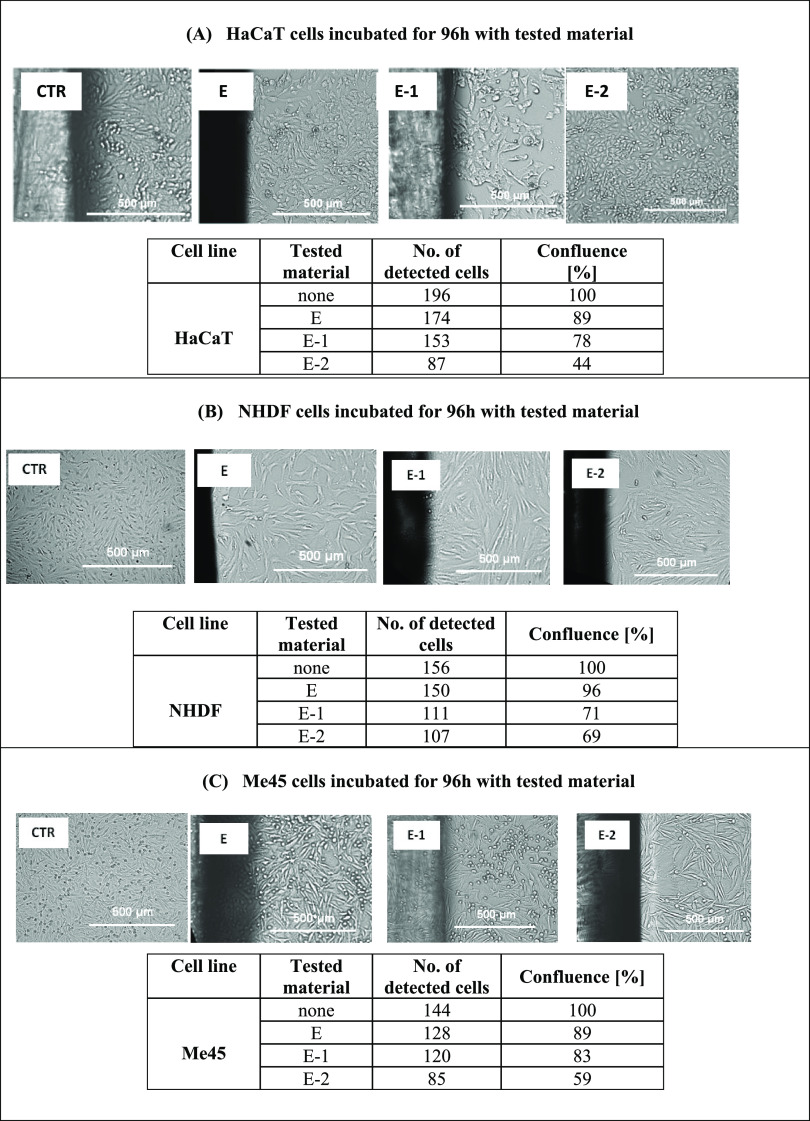
Automated Imaging and Cell Confluence Calculation [%] for Control, Untreated Cells, and Cells Incubated with Tested Materials after 96 h of Incubation with HaCaT Keratinocytes (A), NHDF Fibroblasts (B), and Me45 Cancer Cells (C). CTR, control; E, E+1, and E+2, cells incubated with materials

### 3.8 Cell Proliferation on Material Surface

**Figure 8:**
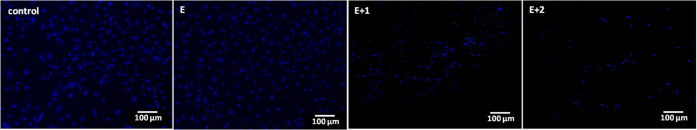
Samples of **c**onfocal images of NHDF cells cultured for 96 h on a control polystyrene plate and tested materials. The nucleus of cells stained with DAPI (scale bar 100 μm); magnification 100×, Olympus FluoView FV1000 apparatus.

The long-term incubation of NHDF cells on the control polystyrene plate and UV-sterilized material surfaces showed good biocompatibilities. The results from confocal imaging (Figure 8) did not differ much from the live observations presented in Table 2. For fibroblasts, the proliferation of materials correlated with the confluence at the materials ages, calculates af confluency parameter, whereas it was 100, 96, 71 and 69% for control, E, E+1 and E+2 samples, respectively. Signals collected by confocal imaging from the material’s surfaces detected nuclei of NHDF cells in all samples (Figure 8). The condition of cells was also manually proved; there were no apoptotic or damaged cells, and most cells were mononucleated (G)/G1 phase, with some binucleated (G2/M) ones, which is a good predictor of the mitosis index. The cells were still able to proliferate on the materials. The images confirm cells’ proliferation abilities, without needing additional cytometric measurements, e.g., for the cell cycle.

### 3.9 Expression of Proinflammatory Cytokines IL6 and IL8

**Figure 9:**
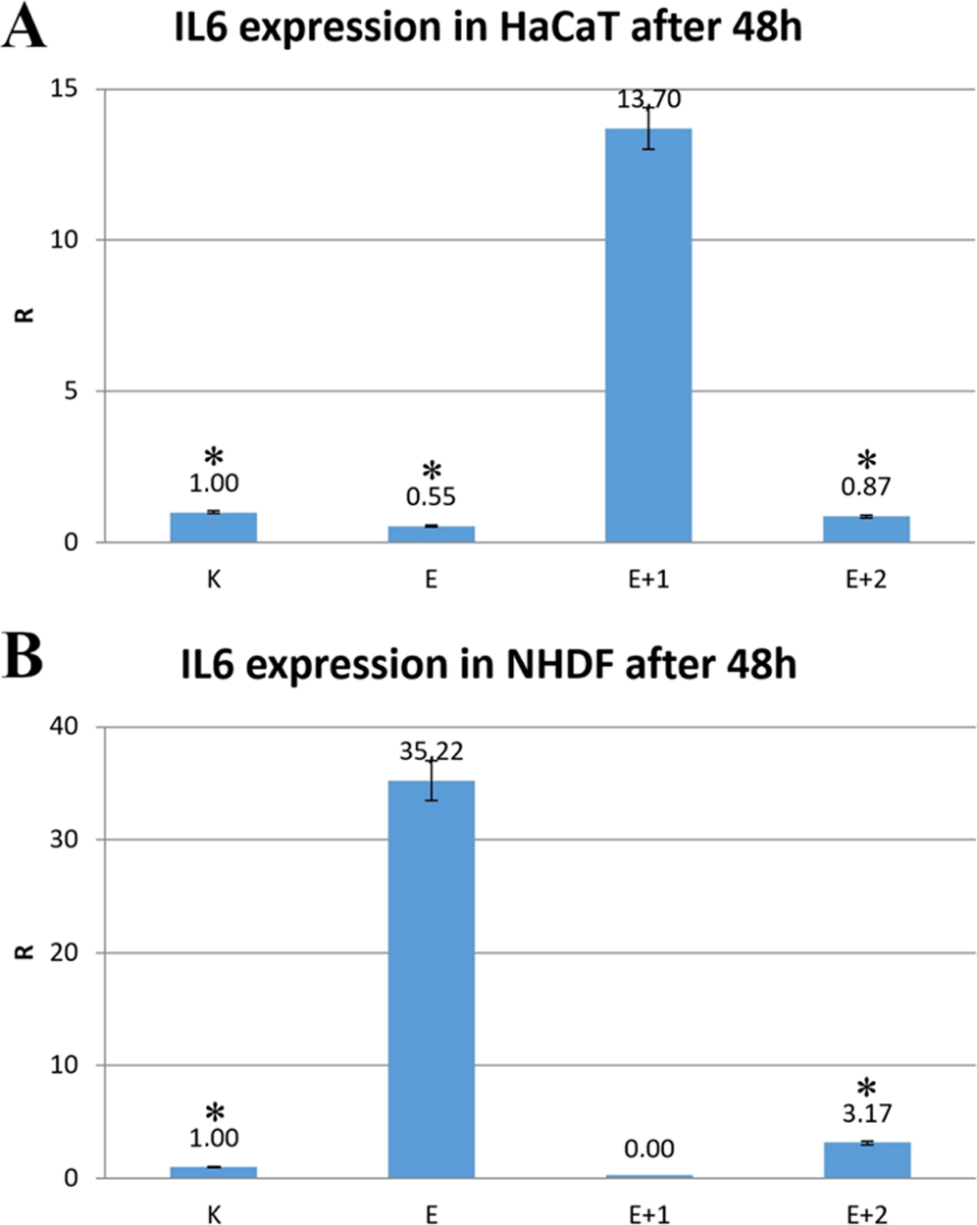
Expression level of the cytokine IL6 in HaCaT (A) and NHDF cells (B) after 48 h of incubation with the materials E, E+1, and E+2. Results were calculated as a Rin reference to the RPL41 gene.

Figure 9A,B presents the results of IL6 and IL8 proinflammatory cytokine expressions for normal HaCaT and NHDF cell lines, whose cultures have been supplemented with the tested materials. The gene expression for these regular skin cell lines was measured after 48 h of incubation.

In HaCaT cells, after 48 h of incubation with the E+1 material, the IL6 gene expression increased 13 times compared to the control. In contrast, samples E and modified E+2 did not induce IL6 expression (Figure 9A). Different trends were observed for the NHDF cells for the same incubation period (Figure 9B). The expression of IL6 increased 35 s more than the control, untreated cells after incubation with the E.IL6 expression was decreased after incubation with E+1 but was slightly elevated after incubation with E+2 (Figure 9B).

The IL6 expression results for two noncancerous skin cell lines differed significantly. High discrepancies in IL6 expression after incubation with E and E+1 should be treated as typical cell response and characteristic for given long-term experiments, where different physiological processes (proliferation, migration, paracrine communications *via* interleukin production, etc.) occur. Analysis of the two independent skin cell lines demonstrated that the tested materials do not tend to induce increased IL6 expression, regardless of the cell line used. Material modification could influence their bioactivity against noncancerous skin cell lines used in live assays. Overexpression of IL6 was not correlated with decreased cell viability and proliferation estimated in MTT and microscopic observation of healthy cells. In such a case, the proinflammatory or anti-inflammatory role of cytokine IL6 is still not apparent. In some research cases materials, such as E and E+1, stimulated IL6 expression, it still results from cells’ proliferation and physiological processes, not confirmed by viability decreasing (by the MTT assay) or proliferation inhibition (microscopic observations) by these materials.

**Figure 10:**
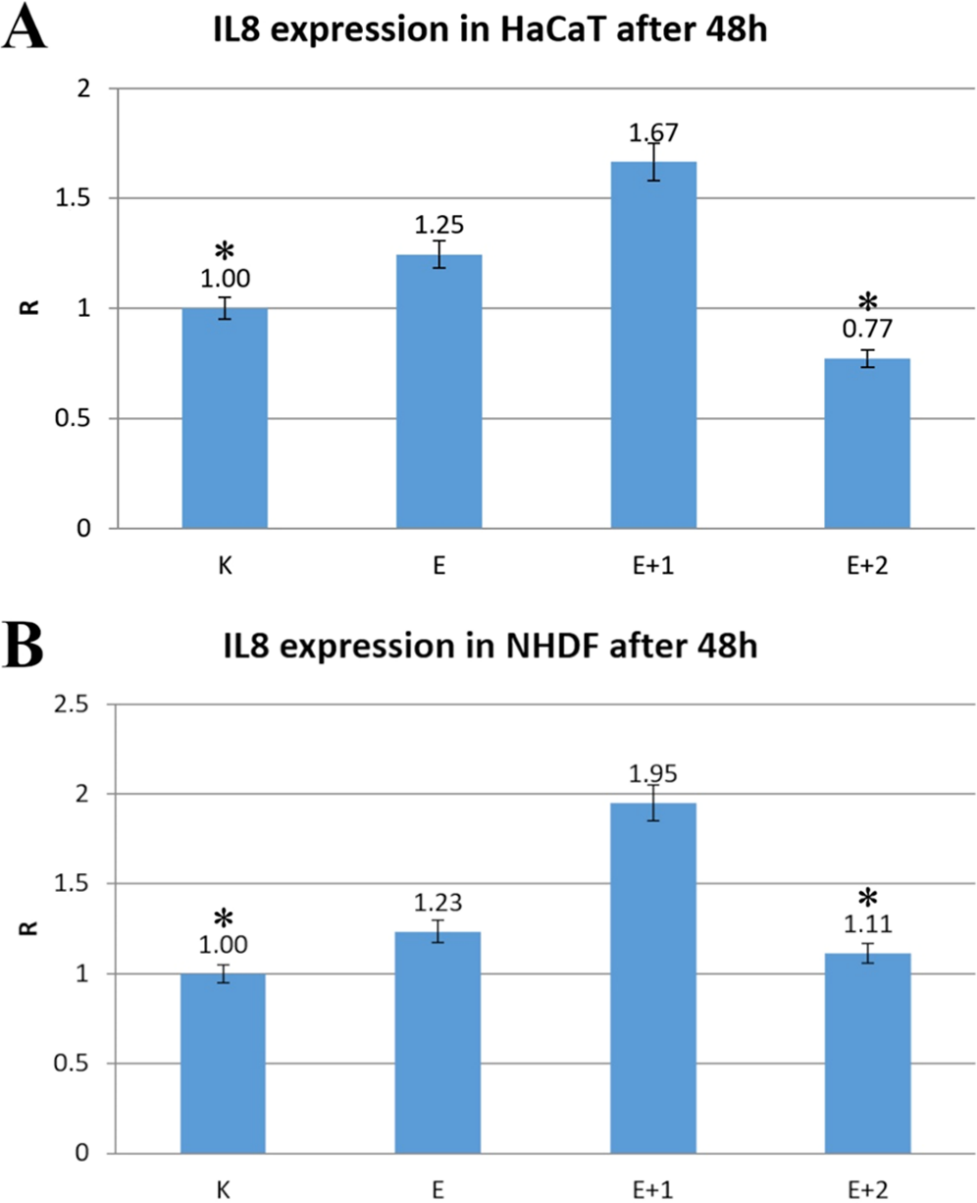
Expression level of cytokine IL8 in HaCaT (A) and NHDF cells (B) after 48 h of incubation with the tested materials E, E+1, and E+2. Results were calculated as R with reference to the RPL41 gene.

Figure 10 shows the results for cytokine IL8 expression in HaCaT (A) and NHDF (B) cell lines, which were cultured with materials E, E+1, and E+2. After 48 h of incubation in both noncancerous skin cell lines, the IL8 gene expression increased by 1.67 and 1.95 times for HaCat and NHDF cells after incubation with E+1 (Figure 10A,B). E and modified E+2 induced little change in IL8 gene expression compared with the untreated controls (Figure 10). The results show no proinflammatory or proapoptotic action in the investigated materials, and no stimulation in proinflammatory cytokine IL8 gene expression was observed in these skin cell lines because viability and proliferation are still comparable to the untreated controls. Such findings predispose modified materials for future on-skin applications. IL8 expression results for cells of two skin cell lines, HaCaT and NHDF, showed similar trends after 48 h of incubation with or without TPU or TPU-HNT. For both HaCaT and NHDF cell lines, IL8 expression values for the material E were very similar, 1.25 and 1.23 times that of the control and untreated cells, respectively. No changes in IL8 expression were reported after incubation with E+2 (Figure 10).

IL6 and IL8 expression studies have shown that none of the tested materials affect the increase in the secretion of these cytokines. This feature may indirectly explain the anticancer effects of the materials tested. The rise in IL6 expression in the body is caused by the inhibition of the tumor necrosis factor (TNF), whose antitumor activity is activated, among others, by inducing apoptosis and differentiation of cancer cells and inhibiting the proliferation of cancer cells. Increased secretion of IL8 may, in turn, result in an increase in the rate of angiogenesis, which is essential in the process of cancer development and metastasis in the human body. The elevated gene expression for E and E+1 materials is a result of physiological processes, whereas no proapoptotic proliferation inhibition of HaCaT or NHDF was observable. The interleukins IL6 and IL8 are also markers of proliferation and healthy cell communication, which could manifest during contact tests with used materials.^62^

## 4 Conclusions

Extruded TPU and TPU filled with HNTs were characterized by AFM microscopy and confirmed to incorporate HNT into the matrix. Using an industrial extruder allowed for an efficient production process of nanocomposites in large volumes. Adding HNT to the polyurethane matrix reduced the contact angle for both the 1 and 2% HNT samples. The results of cytotoxic studies showed a lack of cytotoxicity of the tested materials toward normal, noncancerous skin cells and toxicity toward cancer cells. Further tests showed that the tested materials do not induce the expression of IL6 and IL8 cytokines, whose overexpression may result in cytostatic action (proliferation reduction). This demonstrates that the obtained nanocomposites are characterized by better wettability compared to the parent TPU. Without cytotoxicity toward normal skin cells, they show toxicity to cancer cells.
